# Light weights are as effective as heavy weights for muscle activation in the Hammerobics exercise

**DOI:** 10.1371/journal.pone.0308640

**Published:** 2024-09-17

**Authors:** Koji Murofushi, Tomoki Oshikawa, Koji Kaneoka, Hiroshi Akuzawa, Sho Mitomo, Genki Hatano, Hidetaka Furuya, Kenji Hirohata, Kazuyoshi Yagishita

**Affiliations:** 1 Tokyo Medical and Dental University (TMDU), Sports Science Center, Bunkyo-ku, Tokyo, Japan; 2 Government of Japan Ministry of Education Culture Sports Science and Technology, Japan Sports Agency, Chiyoda-ku, Tokyo, Japan; 3 Waseda University, Faculty of Sport Sciences, Shinjuku-ku, Tokyo, Japan; 4 Niigata University of Health and Welfare, Niigata, Japan; 5 Clinical Center for Sports Medicine and Sports Dentistry, Tokyo Medical and Dental University, Bunkyo-ku, Tokyo, Japan; 6 Institute of Sport Science, ASICS Corporation, Kobe, Japan; 7 Iryo Hojin Shadan Sonodakai, Department of Rehabilitation, Sonoda Third Hospital, Adachi-ku, Tokyo, Japan; Facultad de Ciencias Quimicas, ARGENTINA

## Abstract

**Purpose:**

We previously reported that a Hammerobics exercise using weights can enhance trunk and lower extremity muscles, some studies suggested that training effects could also be expected without heavy weights. If the muscle activity induced by Hammerobics swinging with a ’light plastic ball’ equals or surpasses that of the isometric static squat and synchronized squat with a heavy ball, this training approach could alleviate joint strain, fostering an exercise regimen universally accessible across generations, benefit for workouts sports and rehabilitation.

**Methods:**

Fifteen healthy men participated in this study. By using surface electromyography, muscle activities for the abductor hallucis, tibialis anterior, tibialis posterior, peroneus longus, rectus femoris, biceps femoris long head, semitendinosus, gluteus maximus, multifidus, and internal oblique muscles were measured during a light Hammerobics synchronized squat (HSS-light), Hammerobics synchronized squat, and conventional isometric squat, and statistically compared.

**Results:**

The front-to-back tibialis anterior activity of HSS-light was significantly higher than that of conventional isometric squat. The activities of all other muscles, except for the multifidus, were not significantly different between these exercises in the front-to-back and back-to-front phases. Compared to the Hammerobics synchronized squat, the light Hammerobics synchronized squat showed no differences in front-to-back tibialis anterior, rectus femoris, biceps femoris long head, and internal oblique activities and back-to-front abductor hallucis, tibialis anterior, rectus femoris, and internal oblique activities.

**Conclusion:**

The HSS-light could stimulate muscles to the same level as the conventional isometric squat without weight bearing. While the HSS-light was less effective than the Hammerobics synchronized squat, there was no significant difference in internal oblique, rectus femoris, and tibialis anterior activities between these exercises. Hence, the HSS-light is an exercise method that can be viable approach to promoting accessible workouts sports and rehabilitation.

## Introduction

Perturbation-based exercises, known for their trunk stabilization exercise, extend their positive influence across sports, exercise, and rehabilitation domain. For example, perturbation exercise reduces low-back pain, increases muscle strength and trunk stiffness, and improves trunk stabilization [1]. It can also improve adolescents’ trunk muscle strength and reduce strength imbalances between the flexor and extensor muscles, significantly reducing the prevalence and intensity of low-back pain [2]. Furthermore, perturbation-based trunk stabilization training improves elite rowers’ lower back and physical function via an unstable surface, water-filled pipe, or a push from a third party [3]. Exercise using a water tube can also develop somatosensory/proprioceptive contributions for balance control [4]. Additionally, training using water bags in clean-and-jerk exercises showed higher core muscle activity than training with sandbags and regular barbells [5].

In competitive sports, exercises can be very repetitive and involve the same movement for years, which might increase the risk of an overuse injury [6, 7]. Murofushi developed a perturbation-based exercise called Hammerobics, which requires postural stability and muscle co-activation to avoid the repetitive nature of weight training exercises by oscillating weights from a competition hammer hung on the bar [7]. The Hammerobics exercise was developed from the sport of hammer throw, which is acquired from parametric excitation and can be understood as the hula-hoop model [8, 9].

Murofushi et al. previously measured muscle activities using surface electromyography (EMG) during the Hammerobics exercise. Adding an unstable situation with the hammer’s perturbation swing during an isometric squat significantly activated the activity of the trunk and lower extremity muscles [10]. While this exercise can potentially improve the muscle coordination of the trunk, hip, knee, and ankle joints and develop whole-body coordination, previous results were derived from studies with weights carried on the shoulder and converted from the participant’s body weight range. If effective biological responses can be elicited using significantly lighter weights, this exercise could be employed without imposing stress on the spine and joints. This approach offers potential for diverse generations, promoting accessible workouts across sports and rehabilitation. Therefore, this study aimed to examine potential differences in muscle activities among the conventional isometric squat (CIS), weighted Hammerobics synchronized squat (HSS), and Hammerobics synchronized squat with an extremely light plastic ball (HSS-light), which swings in the anteroposterior direction and has almost no weight. We hypothesized that swinging a light plastic ball would cause some muscles to activate at a nearly identical level to those triggered by the CIS and HSS. The Hammerobics movement, which engages with a swinging motion, requires the reverse movement of the ball, potentially inducing muscle activation, whether using lighter or heavier weights, compared to CIS. Furthermore, based on the principle that a certain weight facilitates easier reversal of movement due to the natural fall of gravity, while lighter weights require more energy for this reversal, we hypothesized that the muscle activation level during the HSS-light exercise would be similar to those observed in HSS. If the HSS-light muscle activity is identical to those of the CIS and HSS, training without putting a strain on the joints will be possible, and this exercise can be offer for diverse generations, promoting accessible workouts across sports and rehabilitation.

## Methods

### Participants

Participants were recruited between June 29, 2021 and April 1, 2022. Fifteen healthy men (mean age, 31.1±6.9 years; mean height, 176.3±7.4 cm; mean body mass, 78.5±15.25 kg; mean BMI, 25.1±3.3 kg/m^2^) who were physically active and exercised at least three days per week, participate in this study. All participants, who had pain on the examination day or severe injuries in the last three months, were excluded. Participants were instructed to stop the experiment when they felt pain during any of the test phases. In this regard, no participants were interrupted due to injury or discomfort during the examination. This laboratory study used a within-participant repeated-measures design. Muscle activity was the dependent variable, and the form of exercise was the independent variable. The study was approved by the Research Ethics Committee of the Tokyo Medical and Dental University (research protocol identification number: M2018-162) and followed the principles of the Declaration of Helsinki (52nd World Medical Association General Assembly Edinburgh, Scotland, October 2000) for medical research involving human subjects. All participants provided written informed consent for study participation. Further, this study is committed to promoting equity, diversity, and inclusion (EDI).

### Protocol

**Fig 1:**
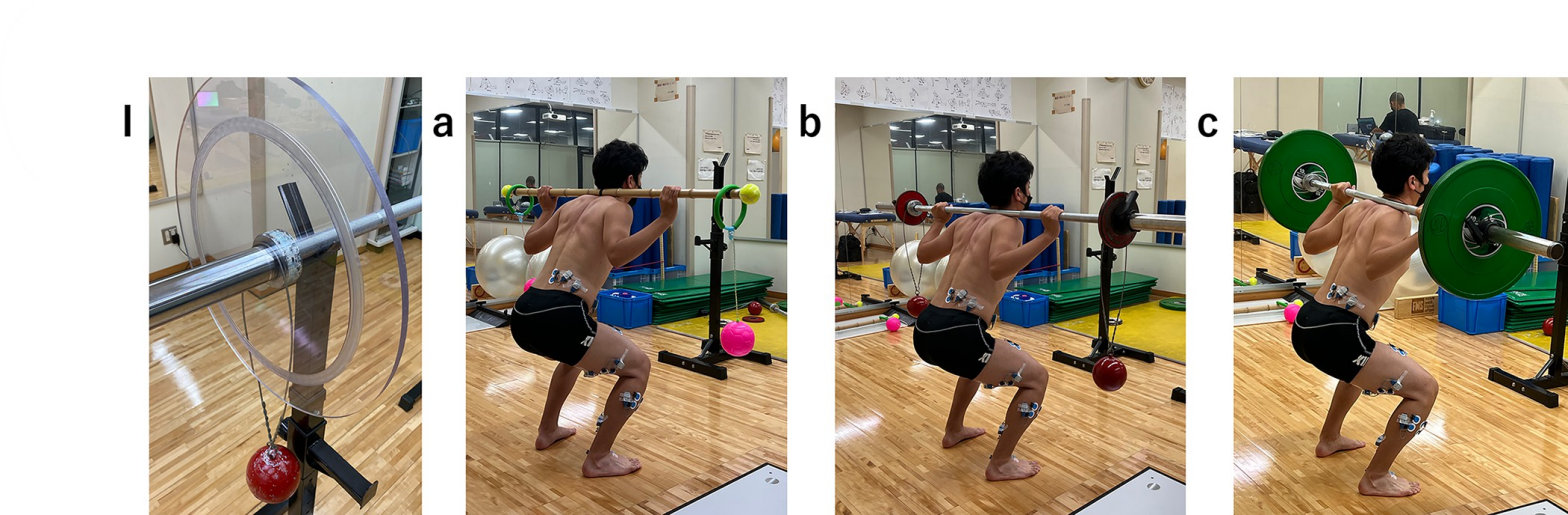
Explanation of the light Hammerobics synchronized squat (HSS-light), Hammerobics synchronized squat (HSS), and conventional isometric squat (CIS). I: Hammerobics Hammer Set Up. **a**: Hammerobics Synchronized Squat-light (HSS-light). **b**: Hammerobics Synchronized Squat (HSS). **c**: Conventional Isometric Squat (CIS).

This study measured EMG data in the muscles of the lower limb and trunk during three exercises. The dominant leg, defined as the leg that kicked the ball, was used as the measurement leg. The exercise tasks were CIS, HSS, and HSS-light (Fig 1). During the exercise, EMG data of the abductor hallucis (Abd H), tibialis anterior (TA), tibialis posterior (TP), peroneus longus (PL), rectus femoris (RF), biceps femoris long head (BFLH), semitendinosus (ST), gluteus maximus (GM), multifidus (Mul), and internal oblique muscles (IO) were measured. All measurements were taken on the same day within the same session. Each exercise were measured twice, in randomized order. Exercises were explained to the participants verbally, and an example of each task was directly demonstrated by an Hammerobics expert, who is an certified athletic trainer. We ensured that an experiment was only performed after sufficient practice to familiarize the participants with the test.

### Data collection and deduction

#### Equipment set-up

**Fig 2:**
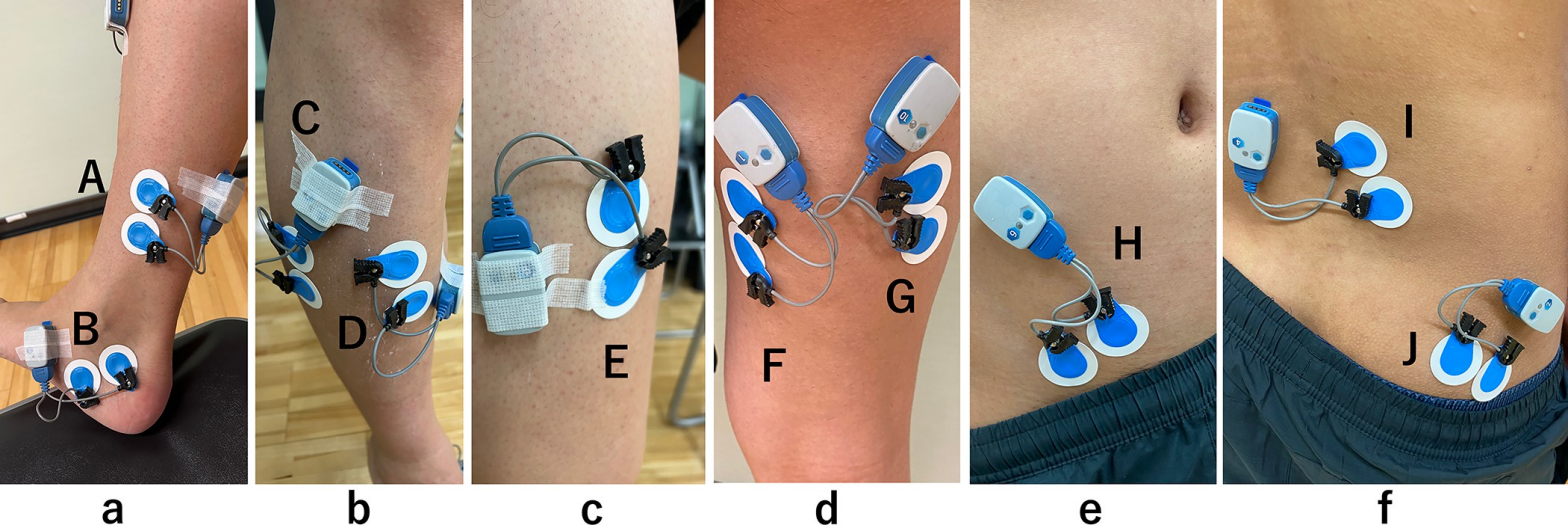
The electrode application site for electromyography. **a**: medial view of the lower leg, **b**: anterolateral view of the lower leg, **c**: anterior view of the upper leg, **d**: posterior view of the upper leg, **e**: anterior view of the abdomen, **f**: posterior view of the lower back. A: Tibialis posterior, B: Abductor hallucis, C: Peroneus longus, D: Tibialis anterior, E: Rectus femoris, F: Semitendinosus, G: Biceps femoris long head, H: Internal oblique, I: Multifidus, J: Gluteus maximus.

EMG signals were recorded during the exercise task using surface EMG (Ultium EMG, EM-U810M8, Tele Myo2400, Noraxon USA Inc., Scottsdale, AZ, USA) and recorded at 2000 Hz with band-pass filtering (10–500 Hz) on a personal computer (EM-P5, Noraxon) using a receiver (EM-U880, Noraxon). The EMG system and the Noraxon Myovideo system using a NiNOX 125 (125 fps, Noraxon USA Inc., Scottsdale, AZ, USA) were synchronized. Prior to electrode attachment, the skin was shaved, abraded, and cleaned with alcohol. The electrode application site for EMG was determined according to previous studies [11–13] and guidelines by SENIAM (http://www.seniam.org/). Surface electrodes (Ambu, Blue Sensor M-00-S, Ballerup, Denmark) were attached 35 mm apart to the Abd H, TA, TP, PL, RF, BFLH, ST, GM, Mul, and IO on the right side (Fig 2). The electrodes for each muscle were attached parallel to the muscle fibers. The skin impedance was confirmed to be <5 kΩ before each measurement [14].

#### Exercise set-up

In HSS, a 7.26 kg hammer (φ: 116.5 mm, NISHI Athletics Goods Co. Ltd., Tokyo, Japan) was hung at each end of the Olympic lifting bar using hammer wire loops. The total length of the equipment from the bottom of the steel ball to the top of the looped wire was 0.5 m. The equipment for the CIS and HSS were set up using the same weight, the total barbell weights were calibrated based on the participant’s individual body weight range as in the previously study: 1) ≤ 110 kg bodyweight: bar (20 kg) + two hammers (7.26 kg each) + two weights (12.5 kg each) = 59.5 kg, 2) 95–109 kg bodyweight: bar (20 kg) + two hammers (7.26 kg each) + two weights (10 kg each) = 54.5 kg, 3) 80–94 kg bodyweight: bar (20 kg) + two hammers (7.26 kg each) + two weights (7.5 kg each) = 49.5 kg, 4) 65–79 kg bodyweight: bar (20 kg) + two hammers (7.26 kg each) + two weights (5 kg each) = 44.5 kg, 5) ≤ 64 kg bodyweight: bar (20 kg) + two hammers (7.26 kg each) + two weights (2.5 kg each) = 39.5 kg [10]. For the CIS, Olympic lifting bar (Men’s Competition Bar WG-157, length: 2200 mm, bar diameter: 28 mm, weight: 20 Kg, sleeve diameter: 50 mm. Uesaka, Tokyo, Japan) and the barbell plates were set by the participant’s individual body weight range. In HSS-light, a 100-g plastic ball (φ: 129 mm) was attached to each end of a 500 g bamboo stick. The total length of the stick was 145 cm, and the 140-cm position was marked as the attachment site of the plastic ball, as in the HSS setup. The total equipment length was the same as in the HSS setup and the radius (from the bottom of the ball to the wire) was the same at 0.5 m. The total weight of the equipment was 353 g. The 100-g plastic ball was chosen for its accessibility and affordability, derived from a commonly available children’s toy.

Before starting the task, the participants had the chance to experience CIS, HSS, and HSS-light to familiarize themselves with these exercises. Participants were instructed to perform the CIS, HSS, and HSS-light exercises with the same body posture. From past studies, knee joint angle during isometric back squat affects muscle activities [15], and a 90° knee angle demonstrated highest muscle activation [15]. In this study we standardized the joint angle at 90° among the three exercises to compare them under same conditions. For each exercise, after placing the bar on the shoulder, the initial stance entails aligning the heels approximately shoulder-width apart while orienting the toes forward or with a slight outward angle not exceeding 10 degrees [16]. After the cue from the examiner, the participant was instructed to slowly crouch. ensuring that the height and knee angle of 90° was maintained during the exercises. Further, a goniometer was used by the examiner to visually evaluate the posture and knee angle before and after the exercise. Each exercise type was performed in two trials, with participants given a 90-second interval between each trial for rest [17].

#### CIS

In the CIS, the participant maintained an isometric squat position to hold the barbell with the same weight as in HSS (Murofushi et al.) [10]. The data was recorded for 10 s during the CIS trial.

#### HSS

HSS was a Hammerobics exercise where both hammers were swung simultaneously (hung with the wire to an Olympic lifting bar) in the anteroposterior direction, followed previous study instructions (Murofushi et al.) [10]. Participants were instructed to slowly crouch and ensure a consistent body height and a knee angle of 90° while swinging both hammers. To ensure safety, the amplitude of the oscillated hammers was kept within 90° of the vertical planes during performance [7].

#### HSS-light

In the HSS-light training, hammers were swung simultaneously in the same direction.

HSS-light was instructed to perform similarly to the HSS in the trials. The exercise created anteroposterior and vertical movements by swinging plastic hammers that were hung via wires at each end of a bamboo stick. Participant were instructed to slowly crouch and ensure a consistent body height and a knee angle of 90° during the exercises while swinging both hammers. During the exercise, the amplitude of the oscillation of the hammers was maintained within 90° of the vertical plane [7]. The main task was to maintain the amplitude and rhythm of the swinging hammers in the anteroposterior direction, during an isometric squat. In both the HSS and HSS-light trials, 10 swings were recorded. Additionally, hammer movements during the HSS-light trial were captured by a high-speed camera that was synchronized with an EMG system.

### Data collection

For the HSS and HSS-light trials, participants gradually swung both hammers until the pendulums became stable and, of the obtained data, the data from three swings were extracted and analyzed. The movements during the HSS and HSS-light trials were divided into two phases of hammer movements, which were captured by the high-speed camera. We defined hammer movements during the HSS as front-to-back (F-B) and back-to-front (B-F). During the F-B phase, the hammer moved from the highest point at the front of the participants to the highest point at the back. For the B-F phase, the movement of the hammer was the opposite of that in the F-B phase, from the highest point at the back to the highest point at the front. EMG data in each phase were used for the analysis. In each CIS trial, we recorded 10 s when the participant was in the initial squat posture. Data between 4.01 and 7.00 s were used.

All raw EMG signals were rectified and smoothed using a root-mean-square algorithm with a 50-ms time reference. This experimental test was not used to compare muscle activities among different muscles. An amplitude comparison of the signals from a given muscle was conducted between the three exercise tasks of an individual in the same session, strictly under the same experimental conditions, and without altering the EMG electrodes [18–20]. The value used for analysis (μV-s) was calculated as the average over the three complete swings during the exercise task.

### Data analysis

Data analyses were performed using IBM SPSS Statistics version 28.0 (IBM Corp., Armonk, NY, USA). The Shapiro-Wilk test was performed to confirm the normality of data distribution. Depending on the normality of the distribution, the one-way analysis of variance (ANOVA) or the Kruskal-Wallis test was used to examine the difference among the exercise tasks and phases. The post-hoc test for one-way ANOVA or Kruskal-Wallis test was the Bonferroni correction. A p*-*value <0.05 was considered statistically significant.

## Results

**Fig 3:**
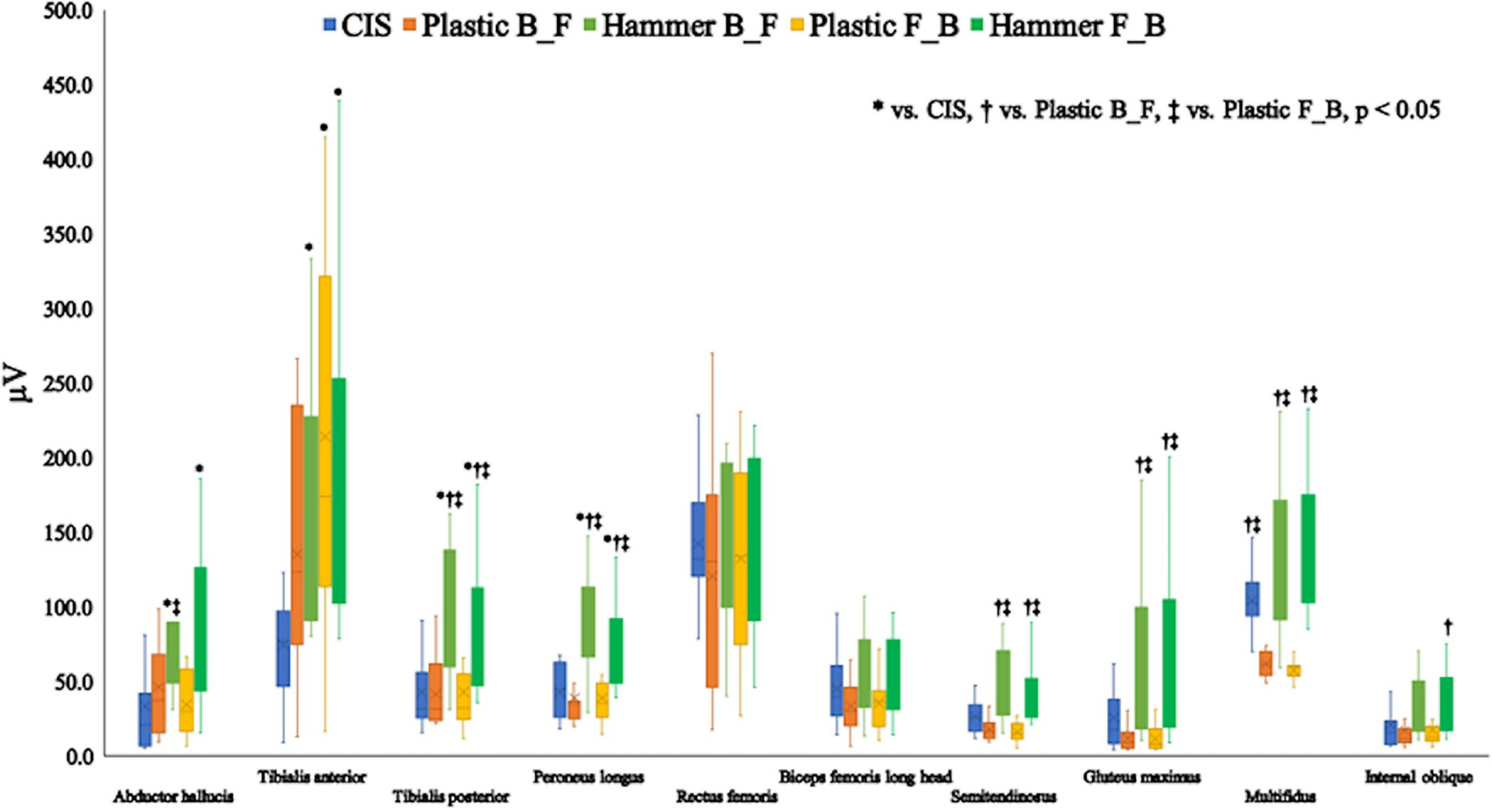
The median and interquartile range of muscle activity during each exercise task. B-F, back-to-front; F-B, front-to-back; CIS, conventional isometric squat, HSS-light hammerobics synchronized squat-light, HSS hammerobics synchronized squat.

Muscle activity level of each muscle in the exercise tasks and phases is shown in Table 1 and Fig 3. Muscle activity of the TA showed significant difference among the exercise tasks and phases (χ^2^ = 21.594, p < 0.001) and HSS B-F (p = 0.003, Cohen’s d = 1.456), HSS-light F-B (p = 0.001, Cohen’s d = 1.583), and HSS F-B (p = 0.001, Cohen’s d = 1.707) demonstrated significantly higher muscle activity compared to CIS. There is a significant difference among exercise tasks and phases in the abd H (χ^2^ = 20.277, p < 0.001). Muscle activities of the abd H in HSS B-F (p = 0.005, Cohen’s d = 1.122), and HSS F-B (p = 0.014, Cohen’s d = 1.083) were significantly higher than that of CIS. Also, HSS B-F showed significantly higher activity than that of HSS-light F-B (p = 0.029, Cohen’s d = 1.196).

**Table 1:** The activity of each muscle (μV-s) in B-F, F-B, and CIS.

Muscle	CIS median (interquartile range)	HSS-light B-F median (interquartile range)	HSS-light F-B median (interquartile range)	HSS B—F median (interquartile range)	HSS F-B median (interquartile range)
Abductor hallucis	20.70 (34.99) *	37.55 (37.68)	29.7 (39.68)	79.25 (31.18)	66.9 (64.85)
Tibialis anterior	76.75 (47.16)	123.55 (124.93)	174 (175.9)	136 (137.00)	199.5 (139.85)
Tibialis posterior	31.65 (27.29)	31.7 (26.23)	32.2 (26.6)	74.35 (75.13)	79.95 (58.98)
Peroneus longus	43.98 (35.02)	34.65 (10.18)	35.35 (18.88)	95.55 (43.28)	86.45 (38.13)
Rectus femoris	132 (41.99)	130.5 (96.4)	134 (95.55)	162 (91.63)	179.5 (81.05)
Biceps femoris	39.54 (27.35)	30.15 (17.93)	36.55 (18.3)	55.35 (31.25)	53.7 (36.35)
Semitendinosus	26.2 (16.32)	16.65 (9.48)	15.35 (9.33)	45 (37.2)	30.95 (22.75)
Gluteus maximus	17.54 (24.60)	9.03 (10.24)	7.94 (11.52)	37.25 (57.48)	36.45 (62.18)
Multifidus	102.73 (18.07)	61.55 (15.5)	59.95 (6.5)	133.5 (64.6)	124.5 (61.25)
Internal oblique	15.5 (14.52)	11.8 (8.57)	12.4 (9.68)	27.25 (28.03)	22.85 (28.6)

B-F, back to front; F-B front to back; CIS conventional isometric squat., HSS-light hammerobics synchronized squat-light, HSS hammerobics synchronized squat

Calf muscle, the TP and PL, demonstrated similar results that there were significant differences among conditions (TP: χ^2^ = 27.065, p < 0.001, PL: χ^2^ = 34.014, p < 0.001). Both muscles showed significantly higher muscle activities in HSS B-F compared to CIS (TP: p = 0.005, Cohen’s d = 1.180, PL: p = 0.002, Cohen’s d = 1.809), HSS-light B-F (TP: p = 0.004, Cohen’s d = 1.215, PL: p < 0.001, Cohen’s d = 1.867), HSS-light F-B (TP: p = 0.004, Cohen’s d = 1.164, PL: p < 0.001, Cohen’s d = 1.911). Similarly, HSS F-B was significantly higher than those of CIS (TP: p = 0.017, Cohen’s d = 1.253, PL: p = 0.028, Cohen’s d = 1.497), HSS-light B-F (TP: p = 0.013, Cohen’s d = 1.313, PL: p = 0.004, Cohen’s d = 1.568), HSS-light F-B (TP: p = 0.014, Cohen’s d = 1.216, PL: p = 0.007, Cohen’s d = 1.616). In the thigh muscles, BFLH and ST showed significant differences among conditions (BFLH: F = 3.182, p = 0.018, ST: χ^2^ = 36.372, p < 0.001). However, Post-hoc analysis for the BFLH revealed that there was no significant difference between conditions. The ST muscle activities in HSS B-F and HSS F-B were significantly higher than those of HSS-light B-F (HSS B-F: p < 0.001, Cohen’s d = 1.687, HSS F-B: p = 0.001, Cohen’s d = 1.494) and HSS-light F-B (HSS B-F: p < 0.001, Cohen’s d = 1.781, HSS F-B: p < 0.001, Cohen’s d = 1.592). There was no significant difference among conditions in RF (F = 2.934, p = 0.569).

Muscle activities of the gluteal and trunks muscles showed significant differences among conditions (GM: χ^2^ = 31.240, p < 0.001, MF: χ^2^ = 52.186, p < 0.001. IO: χ^2^ = 16.067, p = 0.003). The GM demonstrated significantly higher muscle activities in HSS B-F and HSS F-B compared to HSS-light B-F (HSS B-F: p = 0.001, Cohen’s d = 1.282, HSS F-B: p = 0.002, Cohen’s d = 1.218) and HSS-light F-B (HSS B-F: p < 0.001, Cohen’s d = 1.295, HSS F-B: p = 0.001, Cohen’s d = 1.230). Similarly, The Mul showed significantly higher muscle activities in HSS B-F and HSS F-B compared to HSS-light B-F (HSS B-F: p < 0.001, Cohen’s d = 2.021, HSS F-B: p < 0.001, Cohen’s d = 2.308) and HSS-light F-B (HSS B-F: p < 0.001, Cohen’s d = 2.144, HSS F-B: p < 0.001, Cohen’s d = 2.442). Also, muscle activity of the Mul in CIS was significantly higher than those in HSS-light B-F (p = 0.005, Cohen’s d = 2.692) and HSS-light F-B (p < 0.001, Cohen’s d = 2.976). The muscle activity of the IO in HSS F-B was significantly higher compared to HSS-light B-F (p = 0.046, Cohen’s d = 0.826).

**Fig 4:**
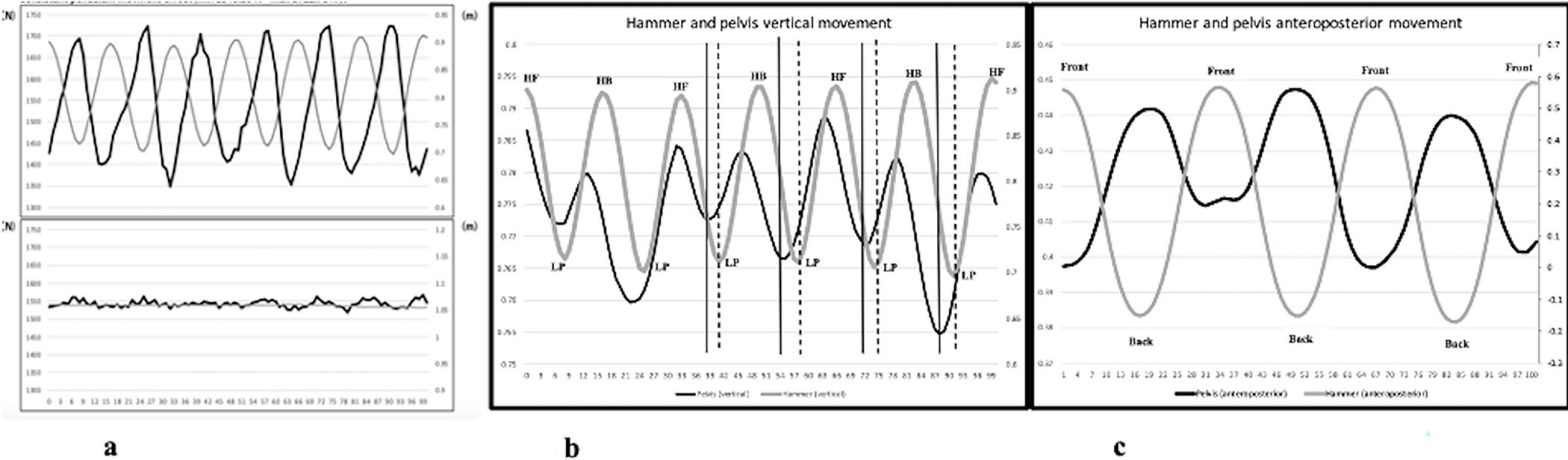
**a**: The relationship between vertical GRF and hammer movement during HSS (upper) and CIS (lower). Range of fluctuation is large for HSS (min 1349.16N ~ max 1722.78 N). **b**: The relationship between the pelvis and hammer movement during HSS (the vertical component). **c**: The relationship between the pelvis and hammer movement during the HSS (the anteroposterior components). Hammer positions indicate as High point Front (HF), High point Back (HB) and Low point (LP) on vertical, Front and Back for anteroposterior.

In Fig 4, a single-case example of the measurement of vertical reaction force, pelvis and hammer movements during the HSS and CIS using a force plate and a three-dimensional motion analysis system illustrated how the body contributed to an active perturbation (subject: 1542 N = 100 kg body weight + 55 kg [55% body weight] = barbell and two 7.26 kg hammers). In Fig 4A (upper panel), we confirmed that vertical ground reaction force (GRF) at the hammer increased at the lowest point and decreased at the highest point, which the practitioner was actively perturbating (i.e., when the hammer was at the lowest point, kinetic energy continued oscillating the hammer). For the CIS, vertical GRF and hammer movement did not change (Fig 4A, lower panel).

In Fig 4B, in the vertical direction, the pelvis was raised before the hammer reached its lowest point, and the pelvis became lower before it reached its highest point. This movement was presumably caused by flexion/extension of the hip, knee, and ankle joints. In Fig 4C, in the anteroposterior direction, the pelvis was in a counter position with the hammer. It could be described as a switching motion to continue swinging the hammer from F-B to B-F phases for power generation by countering the object during swings. Regardless of the presence of weights, this tendency could be expected during Hammerobics.

## Discussion

This study aims to compare muscle activities between swinging a light plastic ball, with heavier wights with CIS and HSS. The Hammerobics movement, which engages with a swinging motion, requires the reverse movement of the ball, potentially inducing muscle activation, whether using lighter or heavier weights, compared to CIS. Furthermore, based on the principle that a certain weight facilitates easier reversal of movement due to the natural fall of gravity, while lighter weights require more energy for this reversal, we hypothesized that the muscle activation level during the HSS-light exercise would be similar to those observed in CIS and HSS. Our study revealed a novel finding that HSS-light muscle activity could stimulate muscles to the same level as the CIS without weight bearing. While the HSS-light was less effective than the HSS, there was no significant difference in internal oblique, rectus femoris, and tibialis anterior activities between these exercises.

In the F-B phase, the HSS-light exercise had higher TA activation than the CIS; there was no difference in the activities of most muscles between the HSS-light and HSS. This suggests that the TA significantly engaged to control with hammer swing, regardless of whether using lighter or heavier weights, compared to CIS. Further, there was no significant difference between the HSS-light and HSS regarding TA, RF, and IO muscle activities in both F-B and B-F phases. No previous reports demonstrated a biological response to hammer swinging exercises with an extremely light weight. The BFLH is not affected by either the weight being carried or the swinging of the hammer in the squat posture. Regarding the Mul, it is believed that they were highly activated to maintain the squat posture while carrying a heavy load, regardless of the hammer swinging.

Perturbation-based training is expected to have various effects. It can strengthen trunk muscles, reduce strength imbalances between the flexor and extensor muscles, and decrease lower back pain [21]. The water-filled training tube elicited a greater level of core muscle activation of the external oblique (EO) and Mul and has also been shown to help develop somatosensory/proprioceptive for sports requiring balance control [4].

Perturbation training includes two concepts of passive perturbation and active perturbation. In passive perturbation, a training effect is created by passively holding the posture under a disturbance controlled by an assistant [22] or an external object, stabilizing the posture created by exercising on an unstable surface [23, 24] or using a waterbag [5]. Alternatively, a perturbation device could be used to evaluate muscle activities while practitioners are challenged to hold the position while overcoming the perturbation [25]. Active or dynamic perturbation is a method where an object actively moves to create a training effect. For example, body-blade is an exercise that uses equipment to actively perturb and strengthen or rehabilitate the shoulder [26]. Further, the Hammerobics exercise can be considered an active perturbation method that utilizes the parametric excitation concept, in which the practitioner proactively swings the object [7, 10].

Recent studies reported that these training effects could be expected even without heavy weights. Schoenfeld et al. analyzed the effects of low- versus high-load resistance training on muscular adaptations for 18 men three times per week for eight weeks of intervention and showed no significant differences between groups and increases in muscle hypertrophy [27]. Morton et al. analyzed resistance-training intervention for 12 weeks in 49 men, in which the higher-repetition group (20–25 repetitions/set) showed 30–50% of 1RM and the lower-repetition group (8–12 repetitions/set) showed 75–90% of 1RM. However, the groups showed no significant differences regarding increases in lean body mass [28].

Regarding non-weight methods for muscle activation, Murofushi et al. reported that muscle activities were enhanced with external focus instructions using paper balloons [29–31]. In the shoulder press task, the external focus instruction was added using the paper balloon with no weights, and muscle activities with 50% and 100% of 1RM were analyzed. Out of 10 muscles in the analysis, five muscles with 50% of 1RM (lower trapezius, latissimus dorsi, medial head of the triceps, pectorals major, and EO) and two muscles with 100% of 1RM (rectus abdominal and IO) were significantly activated [13]. These studies demonstrated that the training effects of light or no-weight training.

In this study, there were no significant differences in the muscle activities of IO, RF, and TA between a light-weight setup (HSS-light) and a heavy-weight setup (HSS) when the practitioners continued the swing actively. For example, if the pelvis was raised before the hammer reached its lowest point, muscles related to the pelvis, knee, and ankle produced a vertical force to continue swinging the hammer.

Depending on environmental or task adaptations, practitioners can incorporate the findings of this study into their programs, offering various exercise options. For instance, post ACL reconstruction or knee surgery, patients are often unable to load weight during rehabilitation. In such cases, HSS-light effectively activates muscles without burdening the surgical limb, serving as a suitable coordination exercise for adolescents or the elderly. It emphasizes minimal body pressure, never exceeding one’s body weight. Additionally, athletes can use this warm-up routine before competition as a neuroadaptation program to efficiently activate specific muscles while minimizing fatigue.

This study had some limitations. First, we only examined dynamic perturbation during HSS and HSS-light and did not analyze passive perturbation during these exercises. Second, during the trials, the lower extremities’ joint angles were not analyzed. However, the knee angle was defined before every trial by an examiner to ensure that the posture did not change during the trial. Third, the order of exercises was not randomized in this study. Moreover, the results here only provided a comparison of the muscle activities, while the effectiveness of the HSS-light exercise was not verified. Forth, since we compared the exercises in the same participant during the same session within a short period, EMG signals were not normalized [22]. These limitations should be addressed in future studies. Lastly, only male participants were included in the study. CIS and HSS exercises require weight settings relative to the participant’s body weight. Heavier participants show a clearer difference between lighter and heavier HSS-light exercises, making the comparison more distinct. Generally, it is easier to find male participants with higher body weights.

In conclusion, the trunk and lower extremity muscle activity in the Hammerobics swinging with a light plastic ball was identical to that in CIS, further it was identical to HSS regarding the IO, RF and TA muscles in this study. Therefore, it will be possible to exercise without strain on the joints, promoting accessible workouts across sports and rehabilitation.

## Supporting information

S1 Checklist STROBE-checklist. (DOCX)
